# A Rare Case Report of Neurodegenerative Disease: Duchenne Muscular Dystrophy in Two Male Siblings

**DOI:** 10.5005/jp-journals-10005-1306

**Published:** 2015-08-11

**Authors:** B Suneja, ES Suneja, VK Adlakha, P Chandna

**Affiliations:** Professor, Department of Pedodontics and Preventive Dentistry, BJS Dental College, Ludhiana, Punjab, India; Reader, Department of Conservative Dentistry and Anatomy, BJS Dental College, Ludhiana, Punjab, India; Reader, Department of Pedodontics and Preventive Dentistry Subharti Dental College, Meerut, Uttar Pradesh, India; Reader, Department of Pedodontics and Preventive Dentistry Subharti Dental College, Meerut, Uttar Pradesh, India

**Keywords:** Disorder, Hereditary, Recessive.

## Abstract

Duchenne muscular dystrophy (DMD) is an recessive X-linked mediated, musculoskeletal disorder that affects only males. It is the most common and severe form of muscular dystrophy where there is failure to manufacture dystrophin. Clinically, it is characterized by progressive muscle wasting eventually leading to premature death. This case report describes the genetic, oral and systemic findings in two cases of DMD in male siblings.

**How to cite this article:** Suneja B, Suneja ES, Adlakha VK, Chandna P. A Rare Case Report of Neurodegenerative Disease: Duchenne Muscular Dystrophy in Two Male Siblings. Int J Clin Pediatr Dent 2015;8(2):163-165.

## INTRODUCTION

Duchenne muscular dystrophy (DMD) is an inherited musculoskeletal disorder that affects only males (recessive X-linked mediated). It is the most common and severe form of muscular dystrophy.^1^ It is characterized by progressive muscle wasting that begins at 3 to 5 years, delay in motor development and eventually wheelchair confinement followed by premature death at about 30 years from cardiac or respiratory complications.

The purpose of this paper is to describe two cases of DMD in male siblings.

## CASE REPORT

**Fig. 1:**
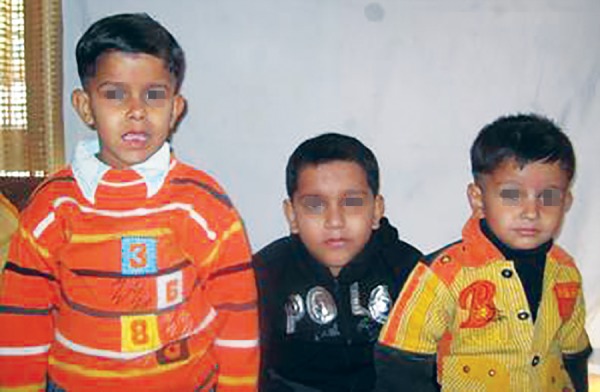
Three male siblings of a family, of which two older children are affected with DMD

Two boys aged 7 and 5 years were brought to a private dental clinic in Meerut, Uttar Pradesh, India for dental treatment. They are a family with three boys (Fig. 1). The two older children aged 7 and 5 years had been diagnosed with DMD at the age of 5 and 3 years respectively. Their parents gave a history of repeated falls, fatigue and inability to climb stairs and muscle weakness for both affected children. Their intelligence quotient (IQ) was in the normal range. The third and youngest child was 3 years old and yet unaffected.

**Fig. 2:**
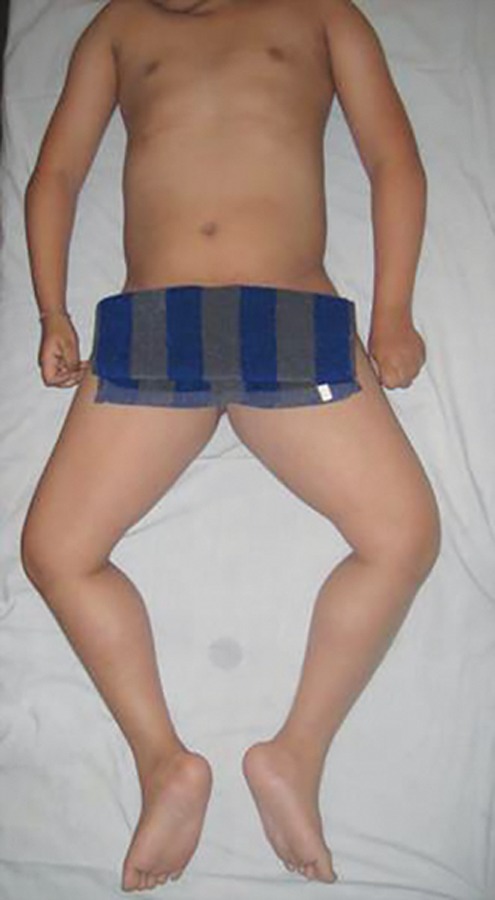
Knee and hamstring contracture in the oldest sibling affected with DMD

**Fig. 3:**
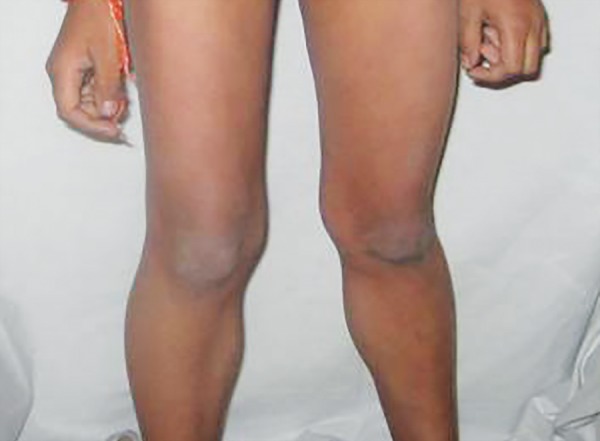
Calf hypertrophy in the middle-ranked sibling affected with DMD

On clinical examination, the oldest child presented with difficulty in standing, walking and stair climbing, obese appearance, proximal weakness, calf hypertrophy, and hamstring muscle contracture (Fig. 2). The other affected child presented with similar difficulty in stair climbing, muscle weakness and calf hypertrophy (Fig. 3). Dental findings in both affected children were open bite and enlarged tongue. Oral prophylaxis was performed for both affected children.

The creatine kinase (CK) levels were elevated to 22040 and 19800 U/L respectively for the oldest and middle child. Electromyographic results were abnormal favoring myopathy for both children. Multiplex polymerase chain reactions (PCR) of the older child revealed dystrophin gene deletion in exons 45, 48, 51, 50, 52 and 60, as seen on agarose gel analysis. For the middle-ranked child dystrophin gene deletion in exons 45, 47, 49 and 50 was present.

Both affected children were undergoing treatment for DMD which included daily physiotherapy, steroid therapy and regular assessment for progressive muscle and cardiac/respiratory damage.

## DISCUSSION

Duchenne muscular dystrophy is the most common muscle dystrophy in India as well as the world.^2^ It is caused by mutations in the dystrophin gene as a result of which the body is unable to synthesize the protein dystrophin that is required for muscle contraction. Each time the muscle contracts, muscle damage occurs, which is repaired – but with the deficient protein. Thus, the repaired muscle is also a damaged one. This continuous cycle of damage and repair and eventually replacement of muscle with fibrofatty tissue is responsible for the clinical signs of progressive muscle wasting and degeneration that is usually apparent by 3 to 4 years.^3,4^ In the present case, the affected older and middle siblings were diagnosed at about 5 and 3 years respectively due to signs of delayed motor development, difficulty in walking and stair climbing, inability to stand from sitting position without support (‘Gower’s sign’), fatigue, repeated falls and muscle weakness.

Oral signs include: wide dental arches, large tongue, delayed eruption, open bite and retrognathic facial morphology. The development of malocclusions in DMD patients is linked to the involvement of the orofacial muscles by the disease.^5,6^ The children in our case presented with enlarged tongue and open bite.

Diagnosis is confirmed by high serum marker levels of CK, genetic analysis or muscle biopsy. Elevated levels of CK in the serum above normal (35-174 U/L) are characteristic in DMD patients. The increased permeability of the sarcolemma that is damaged due to repeated contractions in DMD patients leads to leakage of proteins, such as CK into the plasma.^4,7^ In both affected children in our case, the CK levels were markedly above normal (22040 and 19800 U/L for older and middle siblings respectively).

The underlying cause of DMD is a mutation (most commonly deletions) in the dystrophin gene on the Xp21 chromosome.^8^ The dystrophin gene consists of 79 exons and this is the largest gene in the human genome. Currently, PCR amplification of 19 deletion prone exons (exons 1, 3, 4, 6, 8, 12, 13, 17, 19, 43, 44, 45, 47, 48, 49, 50, 51, 52 and 60) is commonly used to screen deletion mutations.^9^ In our case, deletions of dystrophin gene was seen on exons 45, 48, 51, 50, 52 and 60. Correlation between exonic deletion and certain clinical features like ambulation, mental retardation, and histological findings has been noted.^10^ The children in our case did not suffer from mental retardation.

Current management of DMD involves physiotherapy and corticosteroid therapy which delays but does not cure the disease. Prenatal counseling and various other genetic modalities are being tested to offer hope in this progressive and eventually fatal muscle dystrophy to prolong and improve the quality of life in such patients.
